# Ring finger protein 152-dependent degradation of TSPAN12 suppresses hepatocellular carcinoma progression

**DOI:** 10.1186/s12935-021-01806-1

**Published:** 2021-02-18

**Authors:** Jian Wan, Shunfang Liu, Wanju Sun, Haiyi Yu, Wenlian Tang, Wei Liu, Jing Ji, Bin Liu

**Affiliations:** 1 Department of Emergency and Critical Care Medicine, Shanghai Pudong New Area People’s Hospital, Shanghai University of Medicine and Health Sciences, Shanghai, 201299 China; 2 Jiangsu Key Laboratory of Marine Pharmaceutical Compound Screening, College of Pharmacy, Jiangsu Ocean University, Lianyungang, 222005 China; 3 Department of Oncology, Tongji Hospital of Tongji Medical College, Huazhong University of Science and Technology, Jiefang Road 1095, Wuhan, 430030 People’s Republic of China

**Keywords:** RNF152, TSPAN12, FoxO1, Hepatocellular carcinoma, Ubiquitination, E3 ligase

## Abstract

**Background:**

Hepatocellular carcinoma (HCC) is the third cause of cancer death in the world, and few molecularly targeted anticancer therapies have been developed to treat it. The E3 ubiquitin ligase RNF152 has been reported to regulate the activity of the mechanistic target of rapamycin complex 1 (mTORC1), induce autophagy and apoptosis. However, the relationship between RNF152 and HCC is unclear.

**Methods:**

Transcriptome RNA-sequencing data of HCC samples and normal tissues were used to detect the mRNA expression of RNF152. Luciferase reporter and chromatin immunoprecipitation (ChIP) assays were used to determine the transcriptional regulation of RNF152 in HCC by FoxO1. RNAi, cell proliferation, colony formation and transwell assays were used to determine the in vitro functions of RNF152. Mouse xenograft models were used to study the in vivo effects of RNF152. The immunoprecipitation assay was used to determine the interaction between RNF152 and TSPAN12. The in vivo ubiquitination assay was performed to determine the regulation of TSPAN12 by RNF152.

**Results:**

We found that RNF152 is significantly down-regulated in clinic HCC samples, and its down-regulation is associated with pool overall survival (OS), progression-free survival (PFS) and disease-specific survival (DSS) in HCC patients. The transcription factor FoxO1 was significantly positively correlated RNF152 expression in HCC tissues. FoxO1 recognizes a classic insulin response element (IRE) on the RNF152 promoter to regulate its expression in HCC. RNF152 suppressed HCC cell proliferation, clonogenic survival, invasion in vitro, and tumorigenesis in vivo. Mechanistically, RNF152 interacted with TSPAN12 and targeted it for ubiquitination and proteasomal degradation, thereby inhibiting TSPAN12-dependent CXCL6 expression and HCC progression.

**Conclusion:**

Collectively, our data revealed a tumor suppressor role of RNF152 and a connection between RNF152 and FoxO1 in HCC. Our results support an important role of the FoxO1-RNF152-TSPAN12 axis in the development of HCC. Therapeutic targeting this axis may be an effective means of treating HCC.

## Introduction

Hepatocellular carcinoma (HCC) is one of the top five solid malignancies with the highest incidence in the world, and it is also the third cause of cancer death [1]. There are about 800,000 newly diagnosed cases of HCC each year, and only China accounts for more than 50% [2]. HCC is considered to be the most aggressive tumor and nearly 80% of patients are already at an advanced stage at the time of diagnosis, with considerably poor prognosis. The 5-year recurrence rate after resection is up to 70% [3]. Although current studies have found that the median survival time of patients with advanced HCC who received sorafenib is longer than that of patients who took placebo, there are still many limitations in the current targeted therapy for HCC [4]. Therefore, it is necessary to explore the potential mechanisms that cause HCC tumorigenesis to determine promising HCC therapeutic targets.

Ubiquitination plays an important role in numerous cellular processes and tissue development by affecting the stability, positioning, and function of modified proteins, and has become a universal regulatory mechanism in the process of life [5]. The unbalanced regulation of this process is usually an important factor in the development of cancer [6]. Protein ubiquitination is a multi-step cascade reaction process that requires the participation of at least three enzymes: E1 ubiquitin activating enzyme, E2 ubiquitin conjugating enzyme and E3 ubiquitin ligase, which activate and transfer ubiquitin to the ε-amino lysine residue inside the target protein. The E3 ligase is particularly important because it determines the specificity of the substrates [7]. E3 is divided into two categories according to its protein domain, the HECT domain family and the RING domain family and most E3s members belong to the RING domain family [8]. RNF152 is a typical member of the RING family. It is a single-transmembrane protein, currently found to be mainly located on lysosomes [9]. RNF152 has been identified to be an essential negative regulator of the mTORC1 pathway by targeting RagA for K63-linked ubiquitination in an amino-acid-sensitive manner [10]. Depletion of RNF152 results in the hyperactivation of mTORC1 and protects cells from amino-acid-starvation-induced autophagy [10, 11]. RNF152 has been reported to play a role in tumors and over-expression of RNF152 inhibited colorectal cancer cell proliferation that was dependent on its E3 ligase activity [12]. However, the ubiquitination substrates of RNF152 in tumors is still poorly understood, which seriously hinders the understanding of its biological functions.

In the present study, we found that the FoxO1-dependent down-regulation of RNF152 in HCC tissues is associated with a poor prognosis of HCC patients. Knockdown of RNF152 stimulated HCC cell proliferation, colony formation, invasion in vitro, and tumorigenesis in vivo. Mechanically, RNF152 interacted with TSPAN12 to promote its ubiquitination and proteasomal degradation, thereby inhibiting TSPAN12-dependent CXCL6 expression and HCC progression.

## Materials and methods

### Cell culture and tissue samples

HEK293T, and hepatocellular carcinoma cell lines HepG2, HUH6 and HUH7 cells were purchased from American Type Culture Collection (ATCC). Cells were cultured in high-glucose DMEM (Invitrogen, CA, USA) containing 10% fetal bovine serum (FBS) (Gibco), 100 units/mL penicillin, and 100 mg/mL streptomycin (Gibco) at 37 °C in 5% CO_2_. Twenty paired HCC specimens were obtained from the shanghai pudong new area people’s hospital. The study was approved by the Medical Ethical Committee of shanghai pudong new area people's hospital, and informed consent was obtained from all subjects or their relatives.

### Plasmids and transfection

The cDNAs of RNF152, FoxO1, FoxO3, FoxO4 and FoxO6 were amplified from 293 T or HepG2 cells by polymerase chain reaction (PCR) and cloned into pbabe-Flag vector. All plasmids were completely sequenced and transfected into cells by Lipofectamine 2000 (Invitrogen) according to the manufacturer’s instructions.

### RNA interference, RNA isolation and real-time PCR

The Lentiviral Human RNF152 shRNAs were purchased from Merck (Sigma) and the target sequences for short hairpin RNA (sh-RNA)-expressing plasmids were the following: RNF152-shRNA1:5′CCGGATGTCAGATCTGTTTCAATTACTCGAGTAATTGAAACAGATCTGACATTTTTTTG-3′; RNF152-shRNA2: 5′-CCGGCTTCACAACATGTCTTGCATTCTCGAGAATGC.

AAGACATGTTGTGAAGTTTTT-3′. RNF152-shRNA3: 5′-CCGGGCCCAAGTTGCTGGACTGCAACTCGAGTTGCAGTCCAGCAACTTGGGCTTTTT-3.

TSPAN12-shRNA: 5′-CCGGCATCCGGTCATGATTGCTGTTCTCGAGAACAGCAATCATG.

ACCGGATGTTTTTTG-3′. Total RNA of cell lysate was extracted by using TRIzol reagent (Invitrogen, Shanghai). Oligo dT was used to prime cDNA synthesis. Real-time PCR was then performed by using a SYBR Green Premix Ex Taq (TaKaRa) on Light Cycler480 (Roche, Switzerland) under the following conditions: 95 °C for 3 min, followed by denaturation at 94 °C for 15 s, annealing at 55 °C for 25 s and extension at 72 °C for 15 s for 35 cycles. The relative differences in mRNA levels were calculated by 2−ΔΔCT method. GAPDH was used as internal control. Differences in gene expression were calculated using 2−ΔΔCt method. Primers used for qPCR analysis were list as follows: RNF152 forward, 5′-GGAGACCGCATTCCCTTGG-3′; reverse, 5′-AAAACCGATTGGGCATAAGCC-3′. TSPAN12 forward, 5′-TGTGTCTTTGCAGTGCAGGT-3′; reverse, 5′-GGGTGAAAGAGACTCGGTGA-3′. CXCL6 forward, 5′-CCCACTGGCCTCTGATAAAGG-3′; reverse, 5′-ACGCAAAGGTGCATGATTTG-3′. GAPDH forward, 5′-TGTGGGCATCAATGGATTTGG-3′; reverse, 5′-ACACCATGTATTCCGGGTCAAT-3′.

### Western blotting and antibodies

Cells were lysed with lysis buffer (100 mM Tris–HCl, pH 6.8, 100 mM DTT, 1% SDS, 10% glycerol). Proteins were separated by 10–12% SDS-PAGE, and transferred to NC membrane. Membranes were blocked in 5% non-fat milk in phosphate-buffered saline (PBS) for 1 h before incubation with primary antibody overnight at 4 °C. Membranes were washed with and incubated with secondary antibody for 1 h. Primary antibodies used as indicated: anti-Flag M2 (1:4000 dilution, F1804, Sigma), anti-RNF152 (1:1000 dilution, sc-17775, Santa cruz, USA), anti-TSPAN12 (1:500 dilution, PA5-80195, Invitrogen, USA), and anti-β-Actin (1:5000 dilution, #5174, Cell Signaling Technology, USA).

### Immunoprecipitation (IP)

The IP procedure has been described previously [13]. Briefly, cells were lysed with IP buffer (100 mM NaCl, 20 mM Tris–cl PH8.0, 0.5 mM EDTA, 0.5% (v/v) Nonidet P-40) with protease inhibitor cocktail and phosphorylate inhibitor for 30 min on ice. Cells were sonicated and the lysates were centrifuged. The supernatant was incubated with appropriate antibodies and protein A/G beads overnight at 4 °C in a rotating wheel. Immunoprecipitates were washed eight times with IP buffer. SDS loading buffer was then added and proteins were eluted by boiling at 95 °C for 5 min.

### In vivo ubiquitination assay

Tandem Ubiquitin Binding Entity 2 (TUBE2) resin (LifeSensors) was used to capture endogenous ubiquitinated proteins [14]. Cells after transfection were lysed with IP buffer (100 mM NaCl, 20 mM Tris–cl PH8.0, 0.5 mM EDTA, 0.5% (v/v) Nonidet P-40) with protease inhibitor cocktail and phosphorylate inhibitor for 30 min on ice. Cells were sonicated and the lysates were centrifuged. The supernatant was incubated with TUBE2 resin overnight at 4 °C in a rotating wheel. The resin was then washed with IP buffer for eight times and boiled in SDS loading buffer. Boiled samples were separated by 10% SDS-PAGE and subjected to western blot with indicated antibodies.

### Colony formation analysis

HCC cells after diverse treatment were seeded in a six-well plate at a density of 1000/well and then cultured for about 2 weeks. The numbers of colonies containing more than 50 cells were counted by crystal purple staining. Data were shown as mean ± SD of more than three independent experiments.

### Cell invasion assays

HCC cells were cultured in 10 cm dish with serum-free medium for 24 h. Marigel (Corning) was even plated onto the upper chamber of a Transwell insert (Corning, NY, USA). 5 × 104 HCC cells were suspended with 100 μL of serum-free medium and seeded into the top chamber. The lower chamber was incubated with serum-contained medium. 24 h later, the inserts were fixed with 4% paraformaldehyde (PFA) for 30 min. Cells on the top surface of the inserts were removed and migrated cells on the lower surface of the inserts were stained with 0.05% crystal violet. Images of cells on the Transwell membrane were taken with a microscope and cell numbers were counted. Data were shown as mean ± SD of three independent experiments.

### Luciferase reporter and chromatin immunoprecipitation (ChIp) assays

The promoter region of RNF152 gene (2000 bp upstream of TSS) was amplified from the human genomic DNA and inserted into pGL4.15 vector (Promega, Madison, WI, USA). For luciferase reporter assays, HEK293T cells were seeded in 24-well plates and transfected with the indicated plasmids using Lipofectamine 2000 (Invitrogen) for 36 h. Luciferase activity was measured using the Dual Luciferase Reporter Assay System (Promega). The firefly luciferase luminescence data were normalized by the Renilla luciferase luminescence data. For ChIP assay, a ChIP assay kit (Upstate, Billerica, MA, USA) was used according to manufacturer instructions. Briefly, cells were fixed with formaldehyde and DNA was sheared to fragments at 100–500 bp by repeated sonication. The immunocomplexes were precipitated by using antibodies against FoxO1 or normal serum IgG overnight and rotationally incubation at 4 °C. The immunocomplexes were eluted by using beads according to the manufacturer’s instruction.

### ELISA assay

HCC cells after diverse treatment were seeded on 24-well plate for 48 h to collect medium. The antigen–antibody reaction was performed using DuoSet ELISA for human CXCL6 (R&D Systems) according to the manufacturer’s instructions. Streptavidin–HRP-bound samples were reacted with peroxidase (Sumilon), and measured at an absorbance of 490 nm using a plate reader.

### Xenograft assays

Animal study was approved by Animal Care and Use Committee of shanghai pudong new area people’s hospital. 8-week-old male BALB/cA nude mice were purchased from National Rodent Laboratory Animal Resources (Shanghai, China). All mice were kept in a specific pathogen-free facility and housed at 21 °C ± 1 °C with humidity of 55 ± 10%, fed with sterilized food and water, and kept on a 12 h light/dark cycle. 1 × 107 shRNA-con or sh-RNF152-1-HuH6 cells were suspended in 50 µl of DMEM medium, mixed 1:1 with Matrigel (Corning) and injected into the flanks of male nude mice. Tumor sizes were measured by a caliper and calculated using the formula length × width 2 × 1/2. Tumor weights were measured after mice were sacrificed.

### Statistical analyses

All experiments were at least repeated for three times. Data are presented as mean ± standard deviation (SD). Statistical analysis was performed with GraphPad Prism 7.0 software. The differences between groups were calculated using the Student’s t-test or one-way ANOVA using a Tukey post-hoc test. Statistical significance is displayed as *P < 0.05, **P < 0.01, and ***P < 0.001, respectively.

## Results

### RNF152 is down-regulated in HCC, and its downregulation is associated with a poor prognosis for HCC patients

**Fig. 1:**
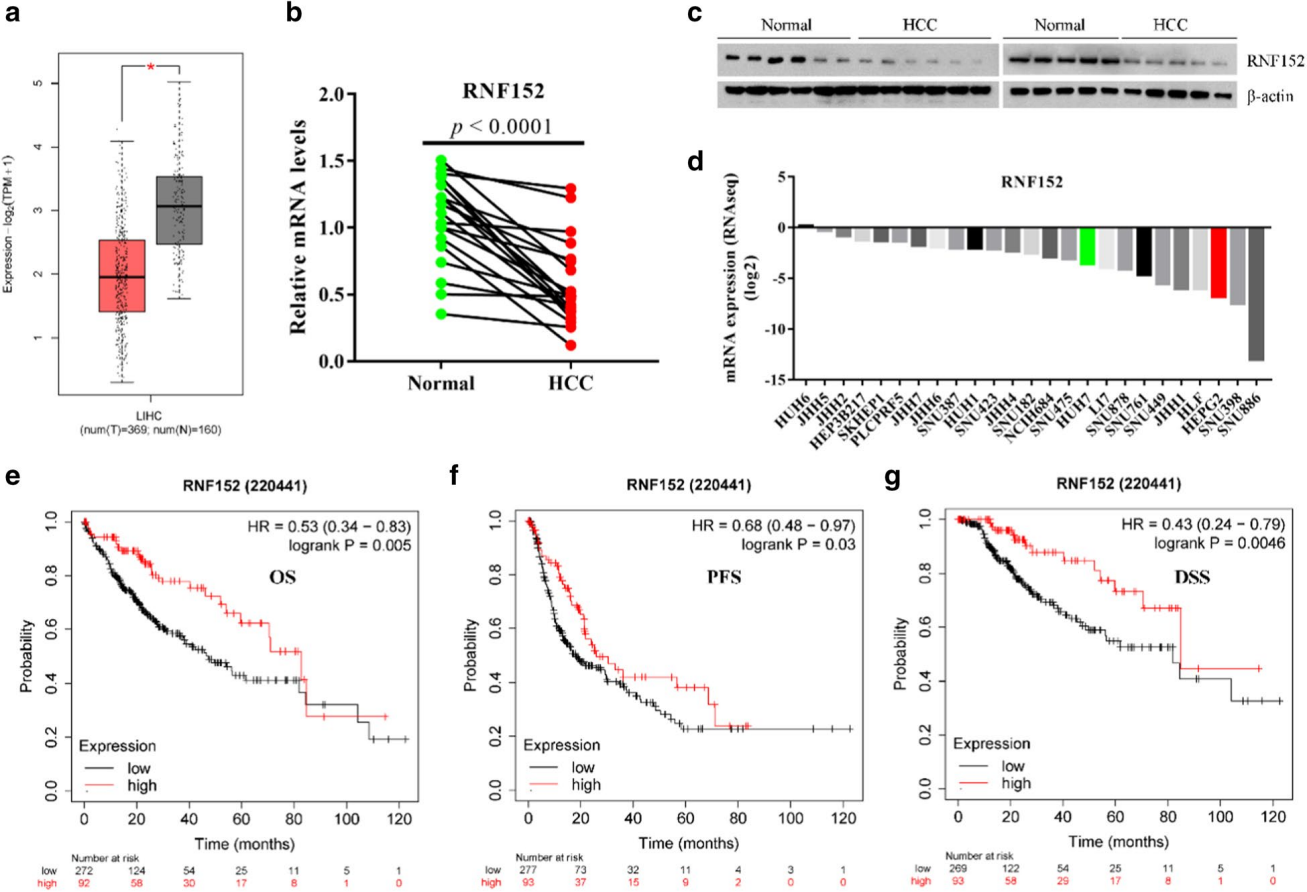
RNF152 is down-regulated in HCC samples and cell lines via FoxO1. **a** RNF152 mRNA expression of 369 tumor and 160 normal tissues in TCGA HCC dataset. **b** RNF152 mRNA expression of 20 fresh paired HCC and adjacent samples were determined by real-time PCR. **c** RNF152 protein expression of 11 fresh HCC and adjacent samples were determined by immunoblotting with indicated antibodies. **d** The mRNA expression of RNF152 in the HCC cell lines in the Cancer Cell Line Encyclopedia (CCLE) database (https://portals.broadinstitute.org/ccle). **e** The overall survival (OS) curve of RNF152 gene expression in patients with HCC in Kaplan–Meier Plotter HCC database (http://kmplot.com/). **f** The progression-free survival (PFS) curve of RNF152 gene expression in patients with HCC. **g** The disease-specific survival (DSS) of RNF152 gene expression in patients with HCC

To investigate the role of RNF152 in HCC, we first analyzed the mRNA expression levels of RNF152 in HCC tissues from the Cancer Genome Atlas (TCGA) database. Compared with 160 cases of the normal tissues, RNF152 mRNA expression was significantly lower in 369 HCC samples (Fig. 1a). We further analyzed RNF152 mRNA expression in 20 fresh paired HCC and adjacent samples and found that the expression of RNF152 in HCC was significantly reduced compared with adjacent normal tissues (Fig. 1b). Consistent with the mRNA level, the protein level of RNF152 was also down-regulated in HCC compared with adjacent normal tissues (Fig. 1c). Next, we analyzed the mRNA expression of RNF152 in the Cancer Cell Line Encyclopedia (CCLE) database (https://portals.broadinstitute.org/ccle) and found that RNF152 was also significantly low expressed in most HCC cell lines (Fig. 1d). To investigate the relationship between the mRNA expression of RNF152 and the prognosis of HCC, we analyzed the online Kaplan–Meier Plotter HCC database (http://kmplot.com/). We found that higher RNF152 expression was associated with better overall survival (OS), progression-free survival (PFS) and disease-specific survival (DSS) in HCC patients (Fig. 1e–g). Together, these results indicate that RNF152 is down-regulated in HCC and low RNF152 level is associated with poor prognosis of HCC patients.

### RNF152 is a transcriptional target of FoxO1 in HCC

**Fig. 2:**
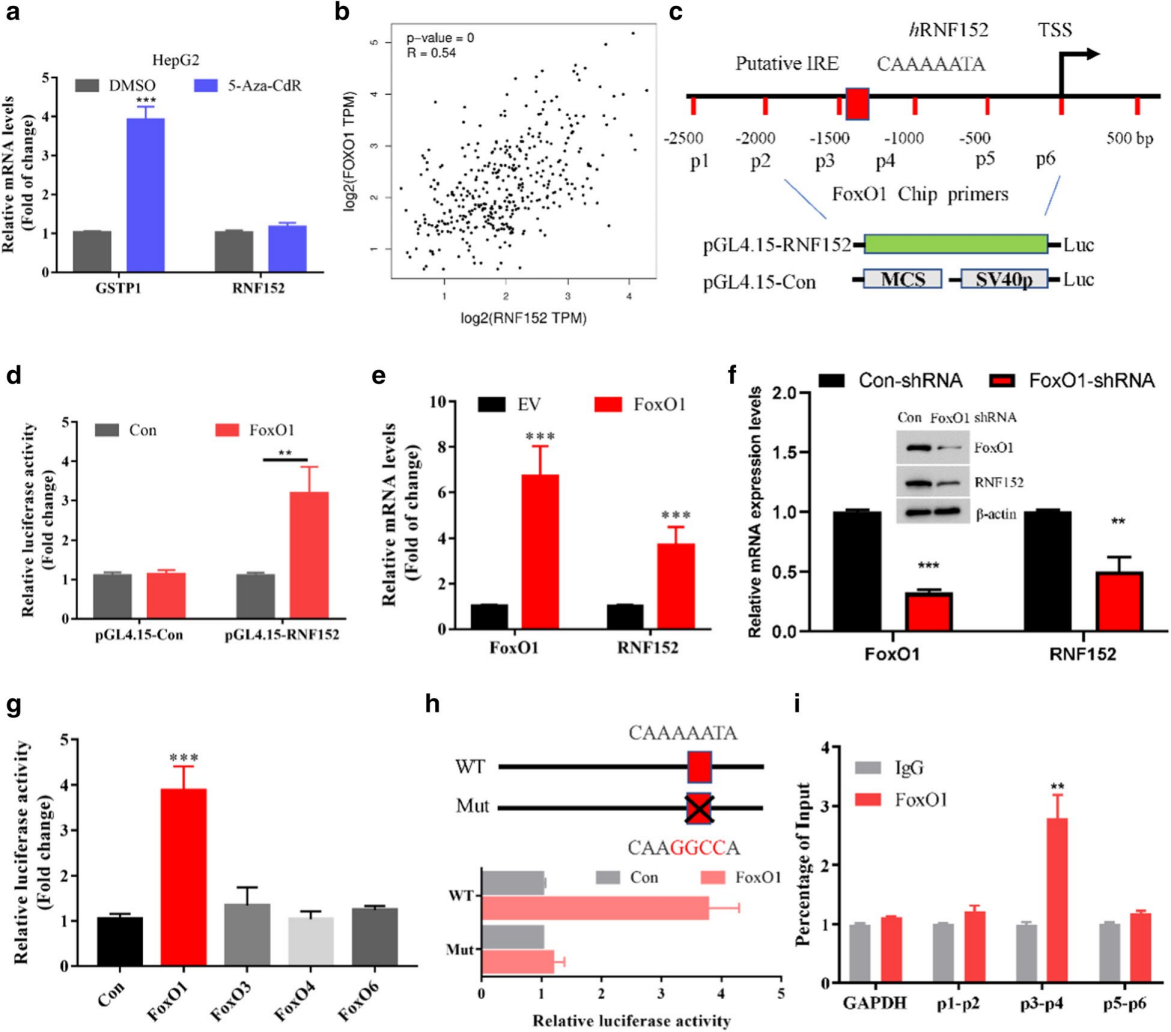
RNF152 is a transcriptional target of FoxO1 in HCC. **a** The mRNA levels of GSTP1 and RNF152 in HepG2 cells with or without 10 μM 5-Aza-CdR treatment were determined by real-time PCR. ***P < 0.001. **b** The correlation between RNF152 and FoxO1 mRNA in the TCGA HCC dataset through the analysis of the GEPIA website. **c** Schematic diagram shows human RNF152 gene promoter and putative FoxO1 binding sites. TSS: transcription start site. IRE: insulin response element. The primer pairs for Chip assay were also listed. **d** pGL4.15-Con or pGL4.15-RNF152 plasmids were co-transfected with either empty vector (EV) or FoxO1 into 293 T cells for 36 h. The luciferase activity was then measured. Overexpression of FoxO1 activated RNF152 promoter-driven luciferase activity. **P < 0.01. **e** HepG2 cells were transfected with EV or FoxO1 for 36 h. The mRNA levels of FoxO1 and RNF152 were determined by real-time PCR. ***P < 0.001. **f** HepG2 cells were transfected with con-shRNA or FoxO1-shRNA for 36 h. The mRNA and protein levels of RNF152/FoxO1 were detected by immunoblotting or real-time PCR, respectively. **P < 0.01, ***P < 0.001. **g** pGL4.15-RNF152 plasmids were co-transfected with indicated plasmids into 293 T cells for 36 h. The luciferase activity was then measured. FoxO1, but not other family members, activated RNF152 promoter-driven luciferase activity. ***P < 0.001. **h** The human RNF152 promoter contains one potential binding sites for FoxO1. Point mutation was highlighted with black cross. FoxO1 was co-transfected with indicated plasmids into 293 T cells for 36 h. The luciferase activity was then measured. Overexpression of FoxO1 activated the luciferase activity driven by RNF152 WT but not RNF152 mutant. **i** ChIP shows enrichment of FoxO1 at the human RNF152 promoter in HepG2 cells. **P < 0.01. The human GAPDH promoter was served as a negative control. The primer pairs for Chip assay were also listed in **c**

Next, we explored the molecular mechanism by which RNF152 is regulated in HCC. The low expression of RNF152 in HCC might be involved the epigenetic regulatory mechanisms, such as DNA methylation. To test this hypothesis, we treated HepG2 and Huh7 cells with 5-Aza-CdR, a DNA methylation inhibitor, respectively. We found that although 5-Aza-CdR treatment can significantly increase the expression of GSTP1, which is known to be epigenetic silenced by DNA hypermethylation in HCC [15]. However, 5-Aza-CdR treatment failed to affect the expression of RNF152 in both cell lines (Fig. 2a; Additional File 1: Fig. S1A), indicating DNA methylation may not be the main reason for the low expression of RNF152 in HCC. We further analyzed the top ten co-expression genes of RNF152 in the TCGA database and found that the transcription factor FoxO1 was significantly positively correlated RNF152 expression in HCC tissues (Fig. 2b; Additional file 1: Fig. S1B). FoxO1 is a known HCC tumor suppressor gene, and its expression is significantly down-regulated in HCC tissues [16, 17]. Therefore, we hypothesized that the low expression of RNC152 in HCC may be related to the low expression of FoxO1. To test it, we first analyzed whether the promoter region of RNF152 contains FoxO1 response elements. FoxO1 recognize two consensus response elements: a Daf-16 binding site (5′-GTAAA(T/C)AA-3′) and an insulin response element (IRE) (5′-(C/A)(A/C)AAA(C/T)AA-3′) [18, 19]. We found that the promoter region of RNF152 contains a putative “5′-CAAAAATA-3′” IRE (from − 1433 to − 1425 bp relative to TSS), which might also be recognized by FoxO1 (Fig. 2c). We then generated a luciferase reporter vector under the control of the RNF152 promoter (− 1500 to + 1 bp of TSS). We found that FoxO1 could induce about 3–4-fold activation of the RNF152 promoter luciferase activity (Fig. 2d). Consistent with it, overexpression of FoxO1 also increased RNF152 mRNA expression of in HepG2 cells (Fig. 2e). Importantly, silencing the expression of FoxO1 by shRNAs resulted in the decrease of RNF152 expression in both HCC and normal human liver cells (Fig. 2f; Additional file 2: Fig. S2C). Thus, these data suggested that FoxO1 can activate RNF152 transcription. On the contrary, other family members including FoxO3, FoxO4 and FoxO6 could not activate the RNF152 promoter luciferase activity as efficient as FoxO1 does (Fig. 2g). Indeed, the correlation of RNF152 with other FoxOs in HCC tissues was less significant relative to FoxO1 (Additional file 1: Fig. S1C–E), further indicating the specificity regulation of RNF152 mRNA expression by FoxO1. As only one potential binding site was existed in the promoter region of RNF152, we then determined whether this site is essential for FoxO1 recognition. We found that mutation of this IRE dramatically blocked the RNF152 promoter luciferase activity driven by FoxO1 (Fig. 2h). Moreover, chromatin immunoprecipitation (ChIP) assays showed abundant occupation of FoxO1 at the RNF152 promoter where the putative IRE located (Fig. 2i). Taken together, these results indicate that FoxO1-mediated RNF152 expression is impaired in HCC samples and cell lines.

### RNF152 acts as a tumor suppressor in HCC

**Fig. 3:**
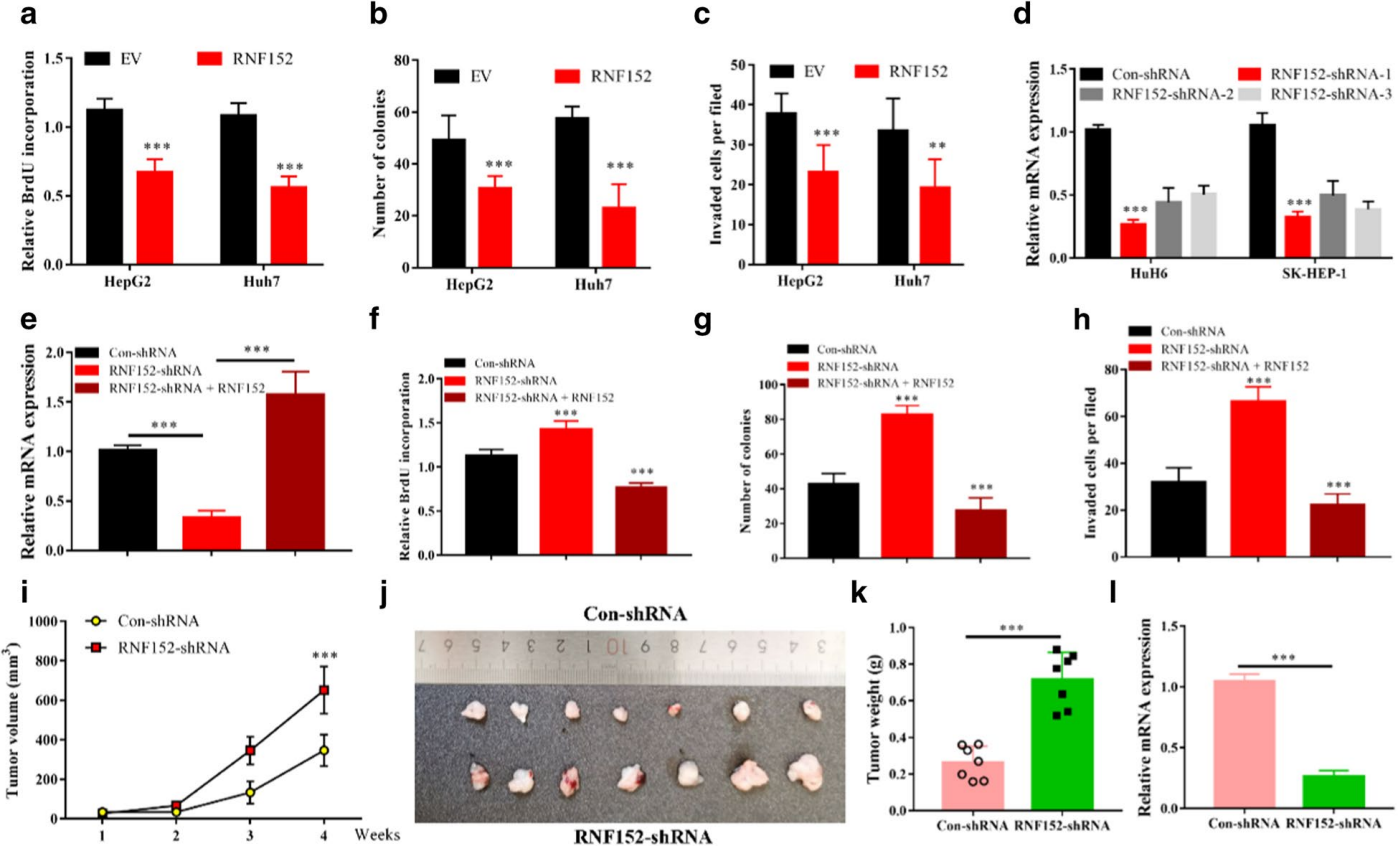
RNF152 acts as a tumor suppressor in HCC. **a** HCC cells with or without RNF152 overexpression were subjected to BrdU test. ***P < 0.001. EV: empty vector. **b** HCC cells with or without RNF152 overexpression were examined for colony formation. *** P < 0.001. **c** HCC cells with or without RNF152 overexpression were examined for cell invasion. ***P < 0.001, **P < 0.01. **d** HCC cells with or without RNF152 silencing (RNF152-shRNA1-3) were examined for FBXO16 mRNA expression by real-time PCR. ***P < 0.001. **e** RNF152-depleted HuH6 cells were transfected with a shRNA-resistant RNF152 plasmid. The RNF152 mRNA levels in these cells were examined by real-time PCR. ***P < 0.001. **f** HCC cells in **e** were subjected to BrdU test. ***P < 0.001. **g** HCC cells in **e** were examined for colony formation. ***P < 0.001. **h** HCC cells in **e** were examined for cell invasion. ***P < 0.001. **i** Each nude mouse was subcutaneously injected with 1 × 10^7^ control HuH6 cells or RNF152-depleted HuH6 cells, and continued observation for 4 weeks. Tumour growth was measured using a caliper at the indicated times after injection. n = 7 for each group. ***P < 0.001. **j** Mice were sacrificed 4 weeks after HCC cells injection. The tumors were then excised, washed with PBS and photographed. K Tumor weights were measured after mice were sacrificed. ***P < 0.001. **l** RNF152 mRNA expression levels in tumors from each group were examined by real-time PCR. ***P < 0.001

Based on the expression levels of RNF152 in HCC cell lines, we generated RNF152 stable overexpressing cells in low RNF152-expressing HepG2 and Huh7 cell lines. The overexpression efficiency of RNF152 was verified at both the mRNA and protein levels (Additional file 2: Fig. S2A, B). Similar to the role of RNF152 in other tumors, overexpression of RNF152 significantly inhibited HCC cells proliferation (Fig. 3a), clonogenic survival (Fig. 3b), and cell invasion (Fig. 3c). To further investigate the biological function of RNF152 in HCC, we used lentiviral-based short hairpin RNA (shRNA) to target the mRNA expression of RNF152 in HCC cell lines. Using three independent shRNA sequences targeting different regions of RNF152 mRNA and two HCC cell lines with relatively high RNF152 mRNA levels (HuH6 and SK-HEP-1), we were able to achieve significant down-regulation levels of RNF152 mRNA. All the three independent RNF152 shRNAs were able to inhibit RNF152 expression, although the knockdown efficiency of RNF152 is somewhat different (Fig. 3d). Among those RNF152 shRNAs, sh-RNF152-1 demonstrated the best inhibitory effect against RNF152 in both HuH6 and SK-HEP-1 cell lines (Fig. 3d), and thus was selected for further experiments. In contrast to RNF152-overexpressing cells, silencing of RNF152 significantly promoted HuH6 cells proliferation, which could be reversed by re-expression of a shRNA-resistant RNF152 (Fig. 3e, f). Moreover, depletion of RNF152 significant promoted clonogenic survival and invasion of HuH6 cells in vitro, which could also be reversed by re-expression of a shRNA-resistant RNF152 (Fig. 3g, h). Furthermore, we then determined the in vivo malignant behaviour of RNF152 in a xenograft mouse model. Nude mice were applied a single subcutaneously injected with either shRNA-con or sh-RNF152-1-HuH6 cells and observed for up to one month. As expected, depletion of RNF152 dramatically enhanced tumorigenesis of HCC (Fig. 3i–l). Together, these data indicate a tumor suppressor role of RNF152 in HCC.

### TSPAN12 was associated with RNF152

**Fig. 4:**
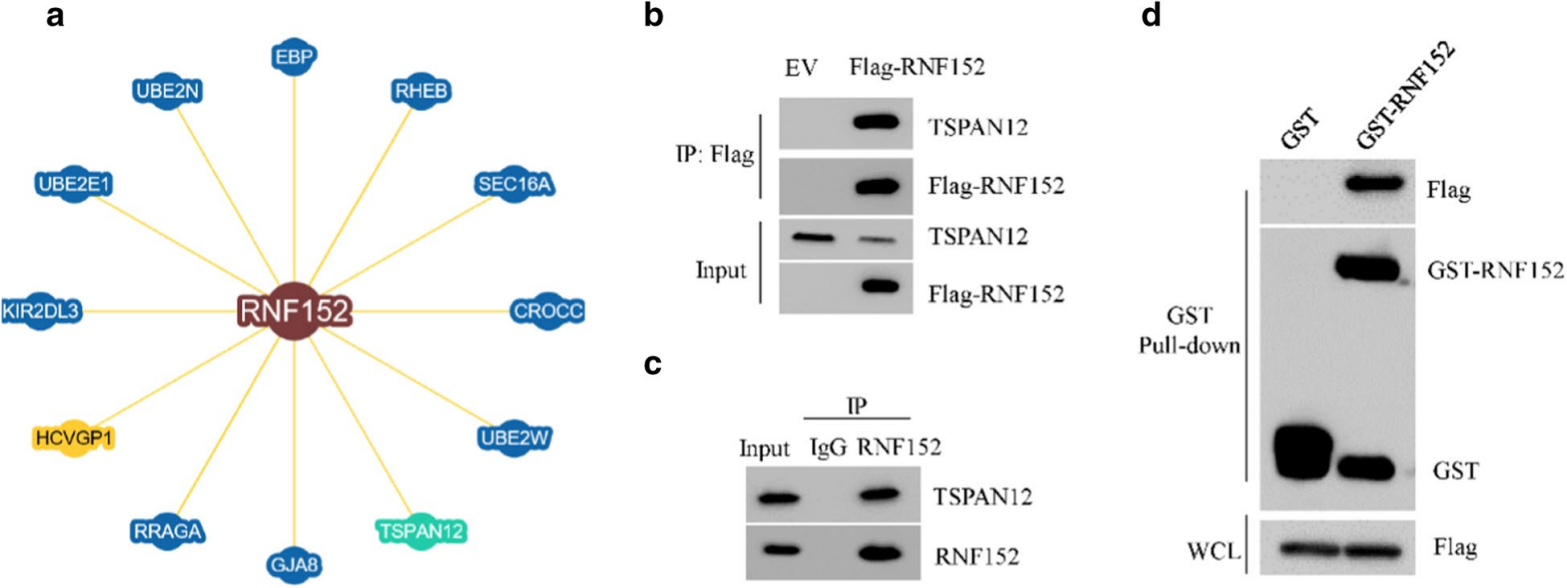
TSPAN12 was associated with RNF152. **a** The interaction protein network of RNF152 revealed by the BioGRID database (https://thebiogrid.org/). **b** The cell lysates from 293 T cells transfected with empty vector (EV) or Flag-RNF152 were subjected to immunoprecipitation (IP) with Flag M2 beads, followed by immunoblotting with indicated antibodies. **c** The cell lysates of MG132-treated HuH6 cells were subjected to immunoprecipitation with IgG or anti-RNF152 antibody and detected by immunoblotting with indicated antibodies. **d** 293 T cells transfected Flag-tagged TSPAN12 plasmid were lysed and incubated with GST alone or GST-RNF152 immobilized on GST-Sepharose beads. The bound proteins were then detected by immunoblotting with indicated antibodies

To better understand the underline mechanisms of the tumor suppressor role of RNF152 in HCC, we searched the interacting proteins of RNF152 through the BioGRID database (https://thebiogrid.org/). A total of 12 proteins have been reported to be associated with RNF152 curated by both high throughput and low throughput. Among these proteins, we found that TSPAN12, which belongs to the tetraspanin protein family [20], is listed as a high throughput associated protein of RNF152 (Fig. 4a). TSPAN12 has been reported to possess oncogenic roles in some kinds of tumors [21–25]. However, unlike RNF152, the mRNA expression of TSPAN12 is not significantly changed in the TCGA HCC cohort (Additional file 3: Fig. S3A) as well in our HCC samples (Additional file 3: Fig. S3B). Moreover, there is no significant correlation between RNF152 mRNA and TSPAN12 mRNA in the TCGA HCC cohort (Additional file 3: Fig. S3C). Thus, we then focused on the relationship between RNF152 and TSPAN12 proteins. To confirm the interaction between RNF152 and TSPAN12, 293 T cells were transfected with Flag-RNF152 for 36 h. Flag-RNF152 protein complex was immunoprecipitated by Flag M2 beads and subjected to immunoblot with anti-Flag or anti-TSPAN12 antibodies, respectively. As expected, TSPAN12 was readily detected in Flag-RNF152 immunoprecipitate (Fig. 4b). Moreover, the interaction between endogenous RNF152 and TSPAN12 was further confirmed in HuH6 cells in the present of the proteasome inhibitor MG132 (Fig. 4c). Furthermore, through GST-pull-down assay, we found that TSPAN12 bound directly to RNF152 protein (Fig. 4d). Together, these data indicate that TSPAN12 is associated with RNF152.

### RNF152 targets TSPAN12 for ubiquitination and degradation

**Fig. 5:**
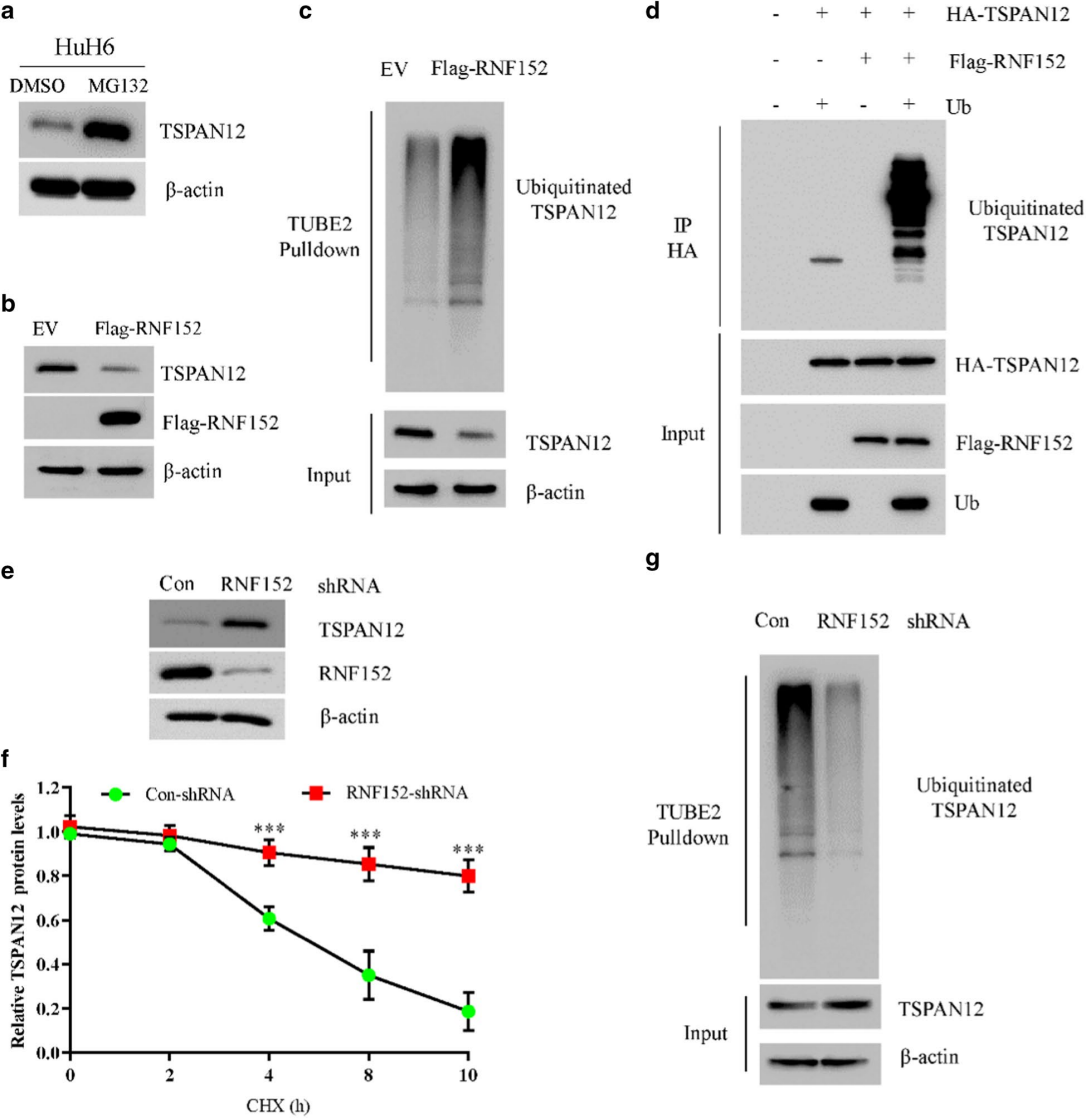
RNF152 targets TSPAN12 for ubiquitination and degradation. **a** The cell lysates of MG132-treated HuH6 cells were subjected to immunoblot with indicated antibodies. **b** The cell lysates of HepG2 cells transfected with EV or Flag-RNF152 were subjected to immunoblot with indicated antibodies. **c** HepG2 cells transfected with control or Flag-RNF152 plasmids were treated with 20 μM MG132 for 4 h. The cell lysates were immunoprecipitated by Tandem Ubiquitin Binding Entity 2 (TUBE2) resin for ubiquitinated proteins enrichment and immunoblotted as indicated. **d** 293 T cells were transfected with the indicated plasmids for 36 h. After immunopurification with anti-HA resin, in vitro ubiquitylation of TSPAN12 was performed in the presence of E1, E2s, Flag-RNF152 and ubiquitin (Ub). Samples were incubated at 37℃ and subjected to immunoblot with indicated antibodies. **e** The cell lysates of HuH6 cells transfected with con-shRNA or RNF152-shRNA were subjected to immunoblot with indicated antibodies. **f** HuH6 cells transfected with con-shRNA or RNF152-shRNA were treated with 40 μM cycloheximide (CHX) for 0, 2, 4, 8, 10 h. The cell lysates were subjected to immunoblot as in **g**. Statistic results of western blotting analysis were obtained by image J software and normalized to actin intensities. Error bars indicate the means ± SD., n = 3. **g** HuH6 cells transfected with con-shRNA or RNF152-shRNA were treated with 20 μM MG132 for 4 h. The cell lysates were immunoprecipitated by TUBE2 resin for ubiquitinated proteins enrichment and immunoblotted as indicated

Because RNF152 is a ubiquitin E3 ligase, the interaction between RNF152 and TSPAN12 led us to speculate that TSPAN12 may be a substrate protein of RNF152. To test this possibility, we first use the proteasome inhibitor MG132 to treat HuH6 and SK-HEP-1 cells, respectively. We found that MG132 can significantly increase the endogenous protein level of TSPAN12 (Fig. 5a; Additional file 4: Fig. S4A), and has little effect on its mRNA level (Additional file 4: Fig. S4B), indicating that TSPAN12 is destructed by the proteasome in HCC cells. Next, we found that overexpression of RNF152 in HepG2 cells caused a decrease in the endogenous TSPAN12 protein level (Fig. 5b), accompanied by increased ubiquitination modifications (Fig. 5c). To test whether RNF152 could ubiquitinate TSPAN12 in vitro, 293 T cells were transfected with HA-TSPAN12 plasmid for 36 h. After immunopurification with anti-HA resin, in vitro ubiquitination of TSPAN12 was performed in the presence of E1, E2s, Flag-RNF152 and ubiquitin (Ub). We found that immunopurified Flag-RNF152 could promote ubiquitination of TSPAN12 in vitro (Fig. 5d). Conversely, knocking down RNF152 resulted in a significant increase of TSPAN12 protein (Fig. 5e). The increased expression of TSPAN12 is due to reduced protein degradation, as the half-life of TSPAN12 is significantly shorten in the absence of RNF152 (Fig. 5f). Consistent with these results, the ubiquitination modification of TSPAN12 was significantly reduced in RNF152-deficient cells (Fig. 5g). Together, these data indicate that RNF152 binds to TSPAN12 and targets it for ubiquitin-dependent degradation.

### RNF152 governed HCC progression partially dependent on TSPAN12 degradation

**Fig. 6:**
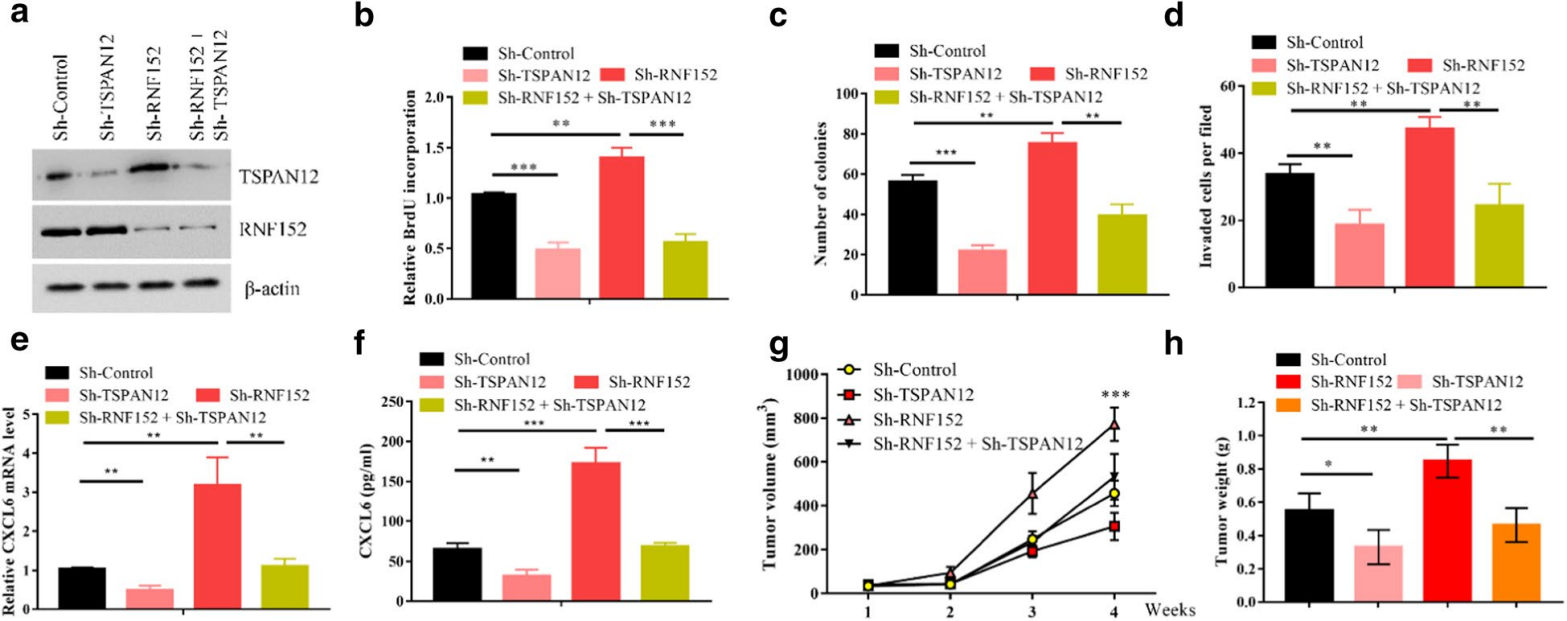
RNF152 governed HCC progression partially dependent on TSPAN12 degradation. **a** HuH6 cells were transfected with or without shRNA against RNF152 individually or simultaneously with TSPAN12. The cell lysates were detected by immunoblotting with indicated antibodies. **b** HuH6 cells in **a** were subjected to BrdU test. ***P < 0.001, **P < 0.01. **c** HuH6 cells in **a** were examined for colony formation. **P < 0.01. **d** HuH6 cells in **a** were examined for cell invasion. **P < 0.01. **e** CXCL6 mRNA expression was regulated by TSPAN12. The mRNA levels of CXCL6 in **a** were determined by real-time PCR. **P < 0.01. **f** The production of CXCL6 secreted from cells in **a** was quantified by ELISA. **P < 0.01, ***P < 0.001. **g** Each nude mouse was subcutaneously injected with 1 × 10^7^ HuH6 cells in **a**, and continued observation for 4 weeks. Tumour growth was measured using a caliper at the indicated times after injection. n = 4 for each group. ***P < 0.001. **h** Tumor weights were measured after mice were sacrificed. **P < 0.01, *P < 0.05

We next investigated whether RNF152 regulates HCC progress through TSPAN12 degradation. Firstly, we stably knocked down both RNF152 and TSPAN12 genes in HuH6 cells by shRNAs. In RNF152 and TSPAN12 double silencing cells, the protein levels of TSPAN12 were close to that of non-specific knockdown cells (Fig. 6a). Inhibiting the expression of RNF152 significantly interfered with the proliferation of HCC cells (Fig. 6b). Silencing of TSPAN12 can partially reverse the acceleration of HCC cell proliferation caused by RNF152 insufficiency (Fig. 6b). Moreover, the enhanced clonogenic survival and invasion abilities of RNF152-depelted cells were largely impaired when TSPAN12 was simultaneously silenced (Fig. 6c, d). TSPAN12 has been reported to promote CXCL6 expression in fibroblasts through the β-catenin signaling pathway, which largely contributed to its oncogenic activity [24]. Indeed, the mRNA and secreted protein levels of CXCL6 were decreased in HCC cells when TSPAN12 was silenced (Fig. 6e, f), suggesting TSPAN12 also regulated the expression of CXCL6 in HCC cells. On the contrary, silencing the expression of RNF152 significantly enhanced the expression and secretion of CXCL6, which could be largely reversed by simultaneously TSPAN12 inhibition (Fig. 6e, f). Thus, these in vitro results suggested a functional interplay between RNF152 and TSPAN12 in HCC involving the CXCL6 signaling pathway. To further test the relation between RNF152 and TSPAN12 in vivo, we established xenografts using those cell lines in nude mice. Our results showed that silencing the expression of TSPAN12 antagonized the enhancement of the in vivo HCC tumor growth induced by RNF152 depletion (Fig. 6g, h). Therefore, these data suggest that the tumor suppressor roles of RNF152 in HCC were at least partly dependent on the degradation of TSPAN12.

## Discussion

In this study, we uncovered a tumor suppressor role of RNF152 in HCC and found that RNF152 is down-regulated and essential to inhibit the proliferation and invasion of HCC via TSPAN12 degradation. Our results demonstrate that RNF152 is a FoxO1 regulated gene in HCC. FoxO1 recognized a putative insulin-response element (IRE) located in the promoter region of RNF152 to induce its mRNA expression. Given the frequently down-regulation of FoxO1 in HCC, the mRNA level of RNF152 was correlated with FoxO1 and significantly decreased in HCC samples. In addition, we analyzed the RNF152 expression in the online Kaplan–Meier Plotter HCC database (http://kmplot.com/), and found that lower RNF152 mRNA level showed marginal unfavorable impact on survival for patients with HCC, indicating a tumor suppressor role of RNF152 in HCC.

RNF152 is a RING ubiquitin E3 ligase, which exerts its biological function by promoting the ubiquitination modification of its substrates. In colorectal cancer (CRC), overexpression of RNF152 has been show to inhibit CRC cell proliferation both in vitro and in vivo by inactivating the mechanistic target of rapamycin complex 1 (mTORC1) via targeting RagA for K63-linked ubiquitination and inducing autophagy and apoptotic cell death [12]. However, it still unknown whether RNF152 could regulate the degradation of its ubiquitinated substrates. Because the E3 ligase must have a direct protein interaction with its substrates, we therefore explored the interacting proteins of RNF152 and identified TSPAN12 as a putative binding partner of RNF152 in HCC.

TSPAN12 plays an important role in regulating cell proliferation, migration and invasion and has been recently shown to be highly expressed in some cancers, such as lung cancer [21], breast cancer [26], ovarian cancer [27] and colorectal cancer [22]. However, the mRNA expression of TSPAN12 is not significantly changed in the TCGA HCC cohort as well in our HCC samples. The inconsistency of TSPAN12 mRNA regulation in HCC samples is most likely due to it being regulated by protein post-translational modification. Indeed, our study found that the protein level of TSPAN12 is regulated by the proteasome pathway. We further revealed that RNF152 can bind to TSPAN12 and promote its ubiquitination-dependent degradation, leading to a decrease in its protein level. TSPAN12 has been reported to promote CXCL6 expression in fibroblasts through the β-catenin signaling pathway [24], which largely contributed to its oncogenic activity. We found that RNF152 could also regulate the expression and secretion of CXCL6 in HCC via TSPAN12. Importantly, silencing the expression of TSPAN12 antagonized the enhancement of the HCC cell growth, colony formation, and invasion in vitro as well as HCC tumor growth in vivo induced by RNF152 depletion. Recently, RNF152 has been identified as a positive regulator of TLR/IL-1R-mediated signaling and overexpression of RNF152 potentiates IL-1β- and LPS-induced NF-κB activation in an ubiquitination-independent manner [28]. Although we and other groups have shown that RNF152 participates in the regulation of tumorigenesis-related processes through its E3 ligase activity, we cannot completely rule out that RNF152 may also participate in some biological processes independent of its E3 ligase activity.

## Conclusion

In summary, we have revealed a tumor suppressor role of RNF152 and a connection between RNF152 and FoxO1 in HCC. Our finding further provides a novel molecular mechanism for the negative regulation of TSPAN12 by RNF152 in HCC cells. RNF152 suppresses HCC progression by ubiquitinating and degrading TSPAN12 to regulate CXCL6 signaling. Our results support an important role of the FoxO1-RNF152-TSPAN12 axis in the development of HCC. Therapeutic targeting this axis may be an effective means of treating HCC.

## Supplementary Information

**Additional file 1: Figure S1**. A The top 10 correlated genes of RNF152 in HCC were identified from TCGA database base on an online analysis website GEPIA2 (http://gepia2.cancer-pku.cn/). B The mRNAs of GSTP1 and RNF152 in Huh7 cells with or without 10 μM 5-Aza-CdR treatment were determined by real-time PCR. *** P < 0.001. C The correlation between RNF152 and FoXO3 mRNA in the TCGA HCC dataset through the analysis of the GEPIA website. D The correlation between RNF152 and FoXO4 mRNA in the TCGA HCC dataset through the analysis of the GEPIA website. E The correlation between RNF152 and FoXO6 mRNA in the TCGA HCC dataset through the analysis of the GEPIA website.

**Additional file 2: Figure S2** A The mRNA levels of RNF152 in HCC cells transfected with EV or RNF152 were determined by real-time PCR. *** P < 0.001. B HCC cells transfected with EV or RNF152 were subjected to immunoblot with indicated antibodies. C Human normal liver cell line HL-7702 cells were transfected with con-shRNA or FoxO1-shRNA for 36 h. The mRNA and protein levels of RNF152 were detected by immunoblotting or real-time PCR, respectively. * P < 0.05, *** P < 0.001.

**Additional file 3:** **Figure S3** A TSPAN12 mRNA expression of 369 tumor and 160 normal tissues in TCGA HCC dataset. B TSPAN12 mRNA expression of 20 fresh paired HCC and adjacent samples were determined by real-time PCR. C The correlation between RNF152 and TSPAN12 mRNA in the TCGA HCC dataset through the analysis of the GEPIA website.

**Additional file 4:** **Figure S4** A The cell lysates levels of MG132-treated SK-HEP-1 cells were subjected to immunoblot with indicated antibodies. B The mRNA of RNF152 in MG132-treated cells were determined by real-time PCR.

## Data Availability

All data generated or analyzed during this study are included in this published article.
