# Supplementary figures and images for: Ring finger protein 152-dependent degradation of TSPAN12 suppresses hepatocellular carcinoma progression
Source: Cancer Cell Int. 2021 Feb 18;21:122. doi: 10.1186/s12935-021-01806-1 (PMC7890835; doi:10.1186/s12935-021-01806-1)

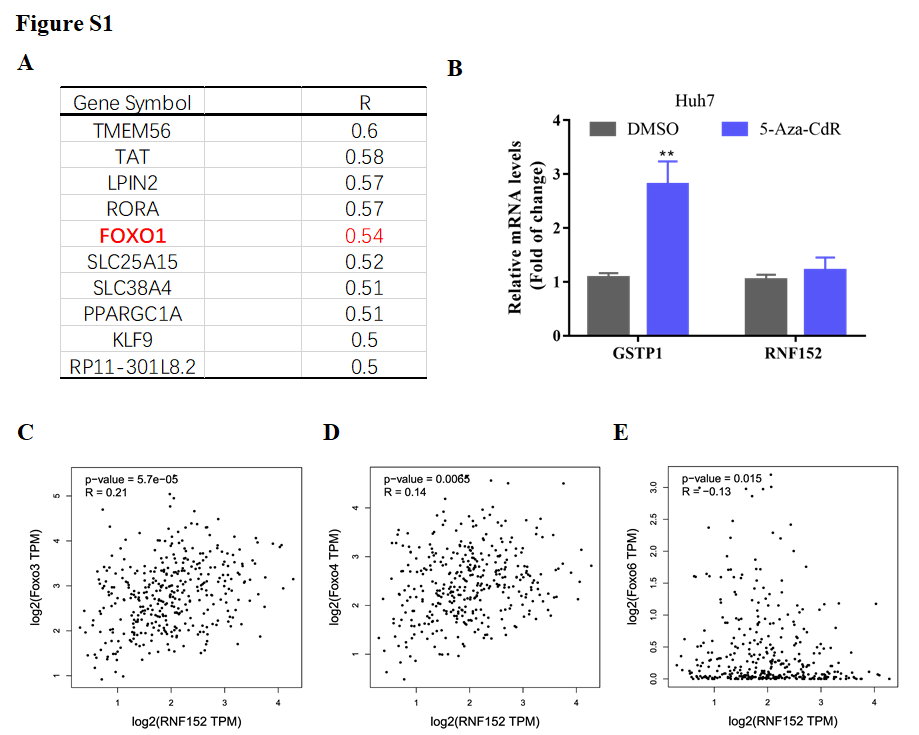

Supplement: Supplementary file 1 — Additional file 1: Figure S1. A The top 10 correlated genes of RNF152 in HCC were identified from TCGA database base on an online analysis website GEPIA2 (http://gepia2.cancer-pku.cn/). B The mRNAs of GSTP1 and RNF152 in Huh7 cells with or without 10 μM 5-Aza-CdR treatment were determined by real-time PCR. *** P < 0.001. C The correlation between RNF152 and FoXO3 mRNA in the TCGA HCC dataset through the analysis of the GEPIA website. D The correlation between RNF152 and FoXO4 mRNA in the TCGA HCC dataset through the analysis of the GEPIA website. E The correlation between RNF152 and FoXO6 mRNA in the TCGA HCC dataset through the analysis of the GEPIA website. [file 12935_2021_1806_MOESM1_ESM.tif]

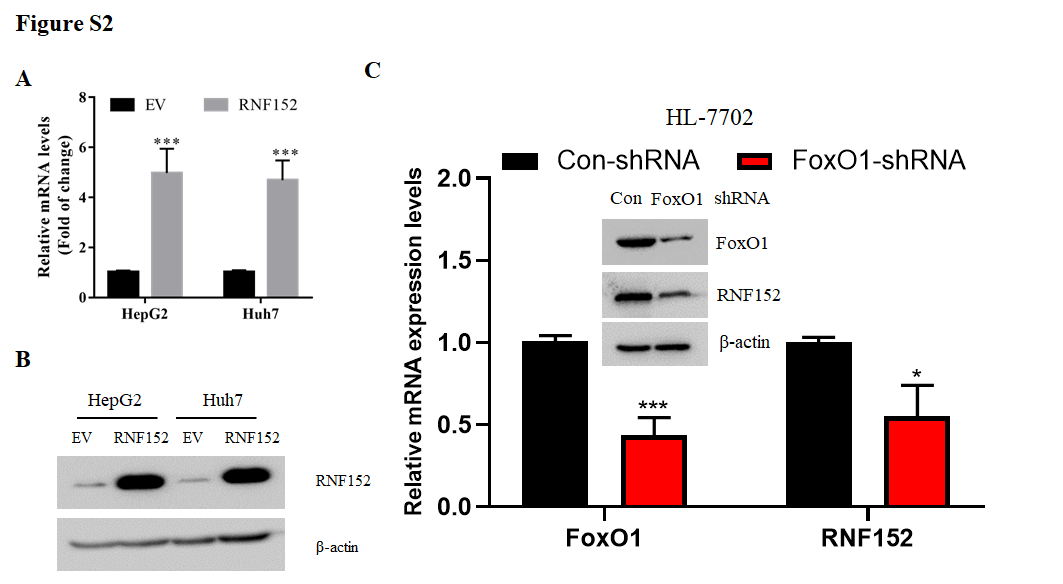

Supplement: Supplementary file 2 — Additional file 2: Figure S2 A The mRNA levels of RNF152 in HCC cells transfected with EV or RNF152 were determined by real-time PCR. *** P < 0.001. B HCC cells transfected with EV or RNF152 were subjected to immunoblot with indicated antibodies. C Human normal liver cell line HL-7702 cells were transfected with con-shRNA or FoxO1-shRNA for 36 h. The mRNA and protein levels of RNF152 were detected by immunoblotting or real-time PCR, respectively. * P < 0.05, *** P < 0.001. [file 12935_2021_1806_MOESM2_ESM.tif]

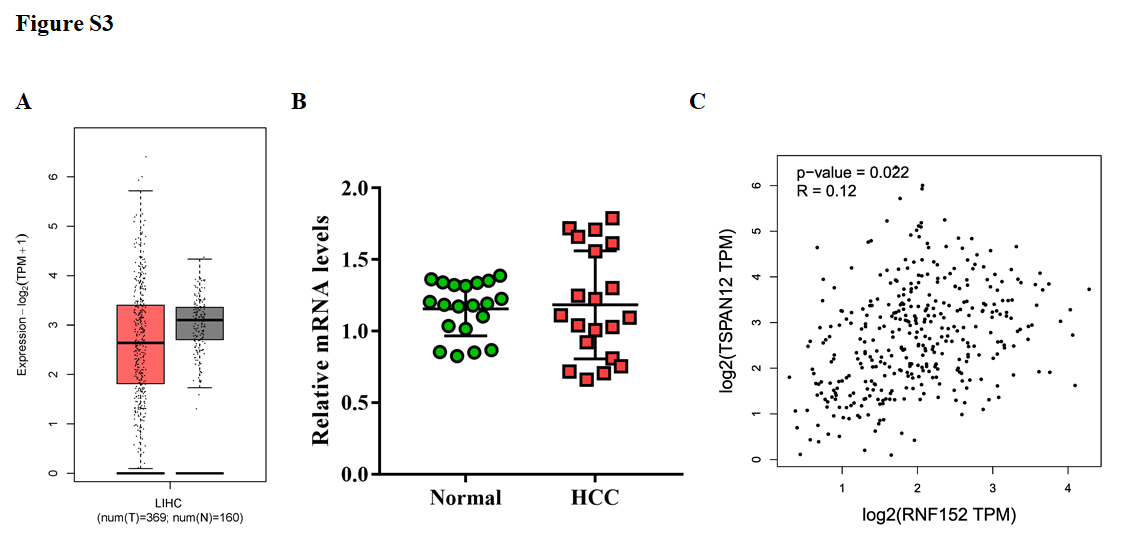

Supplement: Supplementary file 3 — Additional file 3: Figure S3 A TSPAN12 mRNA expression of 369 tumor and 160 normal tissues in TCGA HCC dataset. B TSPAN12 mRNA expression of 20 fresh paired HCC and adjacent samples were determined by real-time PCR. C The correlation between RNF152 and TSPAN12 mRNA in the TCGA HCC dataset through the analysis of the GEPIA website. [file 12935_2021_1806_MOESM3_ESM.tif]

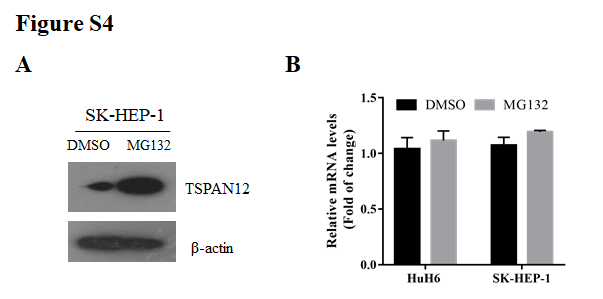

Supplement: Supplementary file 4 — Additional file 4: Figure S4 A The cell lysates levels of MG132-treated SK-HEP-1 cells were subjected to immunoblot with indicated antibodies. B The mRNA of RNF152 in MG132-treated cells were determined by real-time PCR. [file 12935_2021_1806_MOESM4_ESM.tif]
